# Structure of the ordered hydration of amino acids in proteins: analysis of crystal structures

**DOI:** 10.1107/S1399004715015679

**Published:** 2015-10-27

**Authors:** Lada Biedermannová, Bohdan Schneider

**Affiliations:** a Laboratory of Biomolecular Recognition, Institute of Biotechnology CAS, Videnska 1083, 142 20 Prague, Czech Republic

**Keywords:** protein hydration, structural biology, X-ray crystallography

## Abstract

Crystallography provides unique information about the arrangement of water molecules near protein surfaces. Using a nonredundant set of 2818 protein crystal structures with a resolution of better than 1.8 Å, the extent and structure of the hydration shell of all 20 standard amino-acid residues were analyzed as function of the residue conformation, secondary structure and solvent accessibility. The results show how hydration depends on the amino-acid conformation and the environment in which it occurs. After conformational clustering of individual residues, the density distribution of water molecules was compiled and the preferred hydration sites were determined as maxima in the pseudo-electron-density representation of water distributions. Many hydration sites interact with both main-chain and side-chain amino-acid atoms, and several occurrences of hydration sites with less canonical contacts, such as carbon–donor hydrogen bonds, OH–π interactions and off-plane interactions with aromatic heteroatoms, are also reported. Information about the location and relative importance of the empirically determined preferred hydration sites in proteins has applications in improving the current methods of hydration-site prediction in molecular replacement, *ab initio* protein structure prediction and the set-up of molecular-dynamics simulations.

## Abstract 2

The hydration of protein crystal structures was studied at the level of individual amino acids. The dependence of the number of water molecules and their preferred spatial localization on various parameters, such as solvent accessibility, secondary structure and side-chain conformation, was determined.

## 1. Introduction   

Proteins function in an aqueous environment and are evolutionarily adapted to it. Water molecules represent an integral part of protein molecules, their structure, dynamics and function, and understanding of the relationship between the water environment and the polypeptide chain is essential. Water is involved in protein folding (Busch *et al.*, 2013 ▸; Maruyama & Harano, 2013 ▸; Baldwin, 2014 ▸), structure (Takano *et al.*, 2003 ▸; Park & Saven, 2005 ▸) and dynamics (Frauenfelder *et al.*, 2009 ▸; Zhang *et al.*, 2009 ▸; Combet & Zanotti, 2012 ▸) as well as in protein function, such as enzymatic activity (Yang *et al.*, 2004 ▸; Roh *et al.*, 2006 ▸), ligand binding (Ramirez *et al.*, 2008 ▸; Setny *et al.*, 2013 ▸), biomolecular recognition (Reichmann *et al.*, 2008 ▸; Ahmed *et al.*, 2011 ▸) and aggregation (Chong & Ham, 2014 ▸). A range of experimental and computational methods have been used to elucidate the structure and dynamics of the water environment around biomolecules (Chalikian, 2008 ▸). The hydration layer around proteins has been shown to possess physical properties distinct from the bulk water environment; however, the exact parameters (such as the layer thickness and dynamic properties and the extent to which it is structured) are disputed and depend on the method applied and the properties observed (Halle, 2004 ▸; Zhong *et al.*, 2011 ▸). X-ray (Chen *et al.*, 2008 ▸; Kysilka & Vondrášek, 2013 ▸) and neutron diffraction (Niimura & Bau, 2008 ▸) studies provide unique information about the ordered first hydration shell in protein as well as nucleic acid crystal structures. When averaged over many structures, these crystal water molecules consolidate into well defined preferred hydration sites (Schneider & Berman, 1995 ▸; Schneider *et al.*, 1998 ▸; Makarov *et al.*, 2002 ▸; Auffinger & Hashem, 2007 ▸). Information on hydration density, dynamics and residence times can be obtained from NMR experiments (Nucci *et al.*, 2011 ▸), other spectroscopic techniques (Zhang *et al.*, 2007 ▸; Bye *et al.*, 2014 ▸) and molecular-dynamics (MD) simulations (Halle & Persson, 2013 ▸), which can also provide estimates of hydration thermodynamics (Cui *et al.*, 2013 ▸; Hu & Lill, 2014 ▸).

In this paper, we studied the hydration of proteins in crystal structures at the detailed level of individual amino-acid residues. A similar approach has previously been applied in several studies in the 1980s and 1990s (Goodfellow *et al.*, 1993 ▸; Roe & Teeter, 1993 ▸; Flanagan *et al.*, 1995 ▸). Although these studies used only a few crystal structures, they provided valuable information on the preferred water positions around protein functional groups. The observed distributions of water molecules are generally consistent with the stereochemistry of hydrogen bonds and reflect the donor and acceptor properties of the protein atoms. This information has been used in protein structure-prediction software such as *Rosetta* (Jiang *et al.*, 2005 ▸) and *AQUARIUS* (Pitt *et al.*, 1993 ▸). More recently, probability distributions of water molecules around polar protein atoms have been recalculated based on a large number (∼18 000) of protein crystal structures (Matsuoka & Nakasako, 2009 ▸). This analysis confirmed the conclusions of the previous studies, but provided much smoother distributions, allowing more precise predictions (Matsuoka & Nakasako, 2013 ▸). Zheng *et al.* (2013 ▸) calculated radial distribution functions of water around various protein atom types and calculated the corresponding potentials of mean force (wPMF). This allowed the authors to assign a wPMF score to individual water molecules in protein structures and also to predict potential hydration sites.

Considering that a water molecule can simultaneously serve as an acceptor for up to two hydrogen bonds and as a donor for an additional two hydrogen bonds, it is clear that the water position reflects not only the identity of the nearest functional groups but also other groups in its wider binding environment. Therefore, when analyzing the preferred water positions, not only the identity of the amino acid, but also its rotameric state and its environment, such as the secondary structure in which it is located, should be considered (Goodfellow *et al.*, 1993 ▸). When these factors are omitted, the resulting distributions consist of a superposition of different conformational states, leading in some cases to broad distributions that are not structurally interpretable (Matsuoka & Nakasako, 2009 ▸). The conclusion that the first hydration shell is not ordered is then incorrect. The factor of amino-acid conformation has been considered in some of the previous studies (Morris *et al.*, 1992 ▸), in which the main-chain hydration and hydration of serine, threonine and tyrosine have been resolved with respect to the amino-acid secondary structure, although not with respect to the side-chain rotameric state. Beside the neglect of the residue conformation, the available analyses of amino-acid hydration also suffer from too stringent a definition of contacts, for which usually only polar protein atoms (oxygen and nitrogen) are considered, and by considering the side chain and main chain independently. This may lead to the overlooking of unconventional water–protein interactions and of the connection between the main chain and side chains. For example, the nitrogen heteroatoms of tryptophan and histidine side chains have been shown to participate in an off-plane interaction with water (Stollar *et al.*, 2004 ▸), with water–nitrogen distances ranging from 3.0 to 3.4 Å and with an additional coordination of the water to the main chain.

The aim of this study is to analyze hydration around amino-acid residues in protein crystal structures, taking into account the whole residue, its conformation, solvent accessibility and the secondary structure. We test the hypothesis that the first hydration shell is localized in spatially defined hydration sites and that their positions depend on the residue conformation and environment. The protein hydration was analyzed in a set of 2818 crystal structures of sequentially non­redundant, monomeric proteins with a crystallographic resolution of better than 1.8 Å. We investigated the dependence of the hydration of the 20 standard amino-acid residues on their solvent-accessible surface area (SASA), secondary-structure environment and side-chain rotameric state. To structurally interpret the observed differences, we performed conformational clustering of amino-acid residues and Fourier averaging (Schneider *et al.*, 1993 ▸) of water densities and resolved the positions of hydration sites for the main conformational states. The results show how the positions of hydration sites depend on amino-acid conformation and that they can interact simultaneously with side-chain and main-chain protein atoms, and point out unconventional interactions such as carbon–donor hydrogen bonds, OH–π interactions and off-plane interactions with aromatic heteroatoms. These findings can be used to improve current validation protocols and knowledge-based water-prediction programs.

## 2. Materials and methods   

### 2.1. Selection of protein structures   

A set of well resolved protein structures with low sequence homology was selected for the analysis. To this end, the Protein Data Bank (PDB; Berman *et al.*, 2002 ▸) was queried on 27 January 2015 with the following criteria: one protein chain in the biological assembly, chain length of 50–400 residues, resolution of ≤1.8 Å, *R* factor of <0.22, sequence homology of <50%. Numbers of amino-acid residues and water molecules were checked and entries containing no waters were removed.

### 2.2. Preparation of protein structures   

The structures were processed with *REDUCE* (Word *et al.*, 1999 ▸), which is part of the *MolProbity* software (Chen *et al.*, 2010 ▸), in order to correct the flip states of Asn, Gln and His residues and to remove H atoms where present. The ionization states of acids and bases in Arg, Asp, Glu, Lys and His residues, as well as the tautomers of imidazoles (His) and carboxylic acids (Asp, Glu), were not considered in this study. Symmetry-related neighbours of the asymmetric unit were then added by generating the complete content of the unit cell and the cell neighbours. Thus, one unit cell was added in all directions, adding 26 cells surrounding the central cell. For this purpose, a modification of the *GENSYM* program from the *CCP*4 suite (Winn *et al.*, 2011 ▸) was used. The modified *GENSYM* program allows structures with a larger number of atoms to be processed and labels atoms added in neighbouring cells for easier processing. If the asymmetric unit contained more than one protein chain, only the first one was selected for the analysis. In case of atoms with alternate locations, only the ‘A’ position was taken into account. Atoms of the selected protein chain from the unit cell and water molecules from all cells (the unit cell plus the symmetry-generated neighbouring cells) were then extracted for further analysis using *VMD* (Humphrey *et al.*, 1996 ▸).

### 2.3. Distance distributions   

For each amino-acid residue type, distance distributions of water molecules were calculated by counting the number of water molecules within a given distance of any of the residue heavy atoms, processing all amino-acid residues of the given type in the data set. Based on this analysis, a distance cutoff was set for the extraction of individual hydrated amino-acid residues.

### 2.4. Hydration of individual amino acids   

The residue SASA was calculated as the percentage of its surface area accessible to solvent when it was part of the protein chain. The secondary structure of the amino acid was assigned using *STRIDE* (Frishman & Argos, 1995 ▸) within *VMD* (Humphrey *et al.*, 1996 ▸). *STRIDE* assigns each residue to one of the following secondary structures: α-helix, 3_10_-helix, π-helix, extended (β-sheet) conformation, isolated bridge, turn or coil (none of the above). The conformation of the side-chain χ_1_ torsion angle was assigned as follows: 60°, *gauche*+ (g^+^); 180°, *trans* (t); 300°, *gauche*− (g^−^). All conformers were assigned allowing deviations up to ±60°. Individual amino-acid residues were then extracted from the protein structures together with water molecules within the selected cutoff distance.

### 2.5. Conformational clustering   

The extracted amino-acid residues with their surrounding water molecules were aligned with a template residue of the same secondary structure. The secondary-structure class was assigned as described above; only residues within α-helical or β-sheet secondary structure were further analyzed. N, CA, C and CB atoms were used in the alignment for all residues except Gly, in which case N, CA, C and O atoms were used. In each category composed of residues of the same type, secondary structure and *χ*_1_ angle, conformational clustering was performed using the quality threshold (QT) algorithm implemented in *VMD* (Humphrey *et al.*, 1996 ▸). The root-mean-square deviation (r.m.s.d.) of all amino-acid residue atoms was used as the ‘distance function’ for the QT algorithm, with a cutoff value of 0.4 Å. The largest (most populated) cluster in each category, denoted Conformer1, was selected for subsequent analysis.

### 2.6. Hydration sites   

For Conformer1 in each category, the density-representation method (Fourier averaging; Schneider *et al.*, 1993 ▸) was used to find hydration sites as maxima in the water distribution. In order to set an appropriate unit-cell size for the pseudo-electron-density calculation, minimum and maximum coordinates in Cartesian space (*xyz*) were measured for each Conformer1 cluster (all amino-acid residues in the cluster together with all associated water molecules, taking van der Waals radii into account). Distributions of water molecules were then represented as a pseudo-electron density using the *CCP*4 suite (Winn *et al.*, 2011 ▸). This allowed the preferred hydration-site positions to be found as the maxima in the density using the *PeakMax* program from the *CCP*4 suite. Water positions were then ‘refined’ using back-transformed *F*_calc_ values as described in Schneider & Berman (1995 ▸) to obtain the pseudo-*B* factors as a measure of the hydration-site distribution. The procedure was performed in *REFMAC*5 (Murshudov *et al.*, 2011 ▸) from the *CCP*4 suite (Winn *et al.*, 2011 ▸). The significance of individual hydration sites was estimated from their pseudo-occupancies, which were calculated from the local electron density at the hydration-site position after refinement as described in Schneider & Berman (1995 ▸); only hydration sites with a pseudo-occupancy of greater than 0.10 were considered in further analysis unless otherwise stated.

## 3. Results   

### 3.1. Analyzed data set   

The PDB query yielded 2818 protein crystal structures with 587 212 amino-acid residues in the selected protein chains and with 555 667 crystallographically ordered water molecules within 3.2 Å of the selected protein chains. Despite the selection of crystal structures with a relatively high crystallographic resolution, the average water:amino acid ratio of 0.9 within the set of selected chains has a large standard deviation of 0.3.

**Figure 1:**
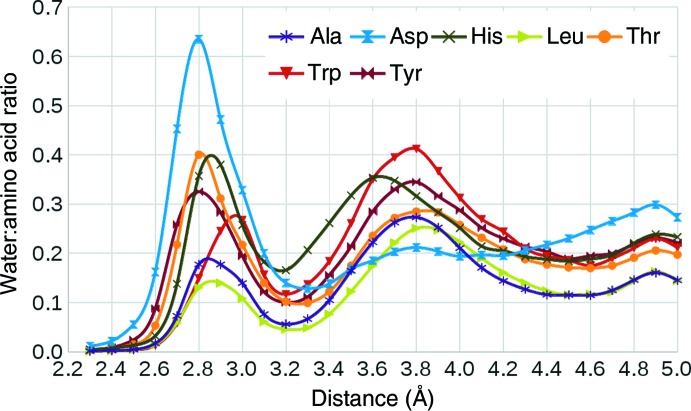
Distance distribution of water molecules around selected amino-acid residues.

In the following, we first discuss the water distance distributions for all 20 standard amino-acid residues (Fig. 1 and Supplementary Fig. S1) and the dependence of the extent of hydration on the solvent accessibility and secondary-structure environment of the residue (Table 1 ▸), and subsequently on the χ_1_ torsion angle (Table 2 ▸). We then describe in greater detail the structure of the first hydration shell of five selected amino-acid residues (Asp, His, Thr, Trp and Tyr), together with the hydration of alanine as a model for the hydration of unhindered main chain (Figs. 3 and 4, Supplementary Figs. S2–S6 and Supplementary Tables S4–S10). The geometries of all 20 amino-acid residues in their main conformational states with their hydration sites are available in the Supporting Information in PDB file format.

### 3.2. Water distance distributions   

Fig. 1 ▸ shows the ratio of waters to amino acids as a function of distance (calculated within 0.1 Å shells) from any heavy atom for the selected amino acids (Ala, Asp, His, Leu, Thr, Trp and Tyr); distributions around all 20 amino acids are shown in Supplementary Fig. S1. In all cases the distribution shows a maximum at around 2.8–2.95 Å corresponding to a hydrogen-bond distance between the water O atom and an amino-acid polar atom. Not surprisingly, the maximum is the strongest for negatively charged Asp and Glu residues and the lowest for hydrophobic residues, which can only form hydrogen bonds through the NH and CO groups of the main chain. The peak appears at a slightly shorter distance (∼2.8 Å) in residues with oxygen in the side chain (Asp, Glu, Thr, Ser and Tyr) than in residues containing nitrogen, with the maximum for Arg and Trp residues lying at about 2.95 Å. Interestingly, the maximum for a His residue lies at ∼2.85 Å, *i.e.* it is shifted towards a shorter interaction distance, probably owing to conjugation of its N atoms to the π-system of the imidazole ring (Dikanov *et al.*, 2007 ▸). Residues containing both nitrogen and oxygen in the side chain (Asn and Gln) show double maxima, with apparently overlapping peaks for hydrogen bonds to oxygen and nitrogen. These observations are consistent with previous studies, in which similar distances have been reported (Thanki *et al.*, 1988 ▸; Kysilka & Vondrášek, 2013 ▸). Most of the hydrophobic residues (Cys, Ile, Leu, Met, Phe and Val) show a similar peak at around 2.9 Å. Interestingly, for Pro, Gly and Ala the peak is higher than for the rest of the hydrophobic residues, likely owing to their small size and good accessibility of the backbone.

The second peak at around 3.6–3.8 Å is broader but is well pronounced for most residues and corresponds to a typical van der Waals (vdW) interaction distance, *cf. *a methane dimer (Takatani & Sherrill, 2007 ▸). This is consistent with the findings of Walshaw and Goodfellow, who reported a maximum in the distance distributions of waters around the CB atom of alanine, the CG1 and CG2 atoms of valine and the CD1 and CD2 atoms of leucine at around 3.8 Å (Walshaw & Goodfellow, 1993 ▸). For some residues (Arg, Lys, Trp, Tyr, Phe and Pro) the amplitude of this vdW interaction peak is comparable to the amplitude of the hydrogen-bonding peak in hydrophobic residues. With the exception of Pro, these are large residues with a combination of hydrophilic end groups and an extensive system of aliphatic (Arg and Lys) or aromatic (Tyr and Phe) CH_*x*_ groups. The distributions shown in Fig. 1 ▸ agree with the results obtained by Chen *et al.* (2008 ▸), who calculated the water–protein radial distribution function for 105 crystal structures and observed two maxima: the first at a radius of 2.75 Å, which they attributed to hydrogen-bond interactions between protein and water, and the second at 3.65 Å, which was attributed to vdW interactions between water and non­polar protein atoms, forming clathrate-like structures.

The third peak, visible for most residues at around 4.9 Å, can be attributed to the second-shell water, in analogy to the second shell in liquid water and ice, which can be observed at a similar distance in both experimental measurements (Finney *et al.*, 2002 ▸; Head-Gordon & Johnson, 2006 ▸) and *ab initio* simulations (Titantah & Karttunen, 2013 ▸). The peak is highest for Asp and Glu residues, suggesting synergy with the strong first-shell peak. Based on the described distance distributions, we selected a cutoff value of 3.2 Å, which corresponds to a minimum separating the hydrogen-bonding and vdW-related peaks for most amino-acid residues. The cutoff was used to extract individual amino-acid residues together with associated waters for further analysis.

### 3.3. Dependence of hydration on residue environment   

For the extracted amino acid, we analyzed how its hydration depends on solvent accessibility and secondary structure (Table 1 ▸). In Table 1 ▸, the residues are grouped in accordance with the classification of Rose *et al.* (1985 ▸) into hydrophobic residues including Ala, Cys, Gly, Ile, Leu, Met, Phe, Pro, Trp and Val, moderately polar residues including His, Ser, Thr and Tyr, and very polar residues including Arg, Asn, Asp, Gln, Glu and Lys. The residues Ala, Leu, Asp, His, Thr, Trp and Tyr, which are discussed in greater detail, are highlighted in bold in Table 1 ▸.

Firstly, we analyzed the dependence of the water:amino acid ratio on the residue SASA within the protein chain from which it was extracted. Interestingly, the difference between hydration at SASA values of 5–30% and SASA values of >30% is quite small, being extremely small in the group of very polar residues (both in relative and absolute numbers) and slightly higher in the group of hydrophobic residues. Moreover, the polar residues retain more than 50% of the hydration even at extremely low SASA values (SASA of <5%). This indicates that the hydrogen-bonded hydration waters are part of the protein and that hydration is characteristic of the residue type rather then its solvent exposure, *i.e.* whether it is solvent-exposed or buried. It also demonstrates the ability of water molecules to penetrate deep into the protein structure, which might be important for the structural integrity of proteins as well as for catalytic function (Williams *et al.*, 1994 ▸; Park & Saven, 2005 ▸; Bottoms *et al.*, 2006 ▸). Because of the relatively small difference between the water:amino acid ratios at various SASA levels, amino-acid residues from all SASA levels were analyzed together in the subsequent analyses.

Secondly, we analyzed the dependence of the water:amino acid ratio on the secondary-structure type in which the residue occurs. The average ratio of the number of water molecules per amino acid (within 3.2 Å) in different secondary structures [α-helix (H), β-sheet (E) and turn (T)] show significantly different patterns for hydrophobic and polar residues (Table 1 ▸). The turns are hydrated the most in all residues, but while the moderately and highly polar residues have a similar water:amino acid ratio in all analyzed secondary structures, the differences are much larger among the hydrophobic residues. Gly and Ala residues behave similarly to most other amino acids and demonstrate the hydration of sterically unhindered protein main chain. An interesting exception is Asp, which is hydrated more in α-helix than in turns. Similarly, Glu, which is just one CH_2_ group longer, is hydrated similarly to all other amino-acid residues: less in α-helix than in turns. This demonstrates that amino-acid hydration is determined not only by the residue type but also by the specific environment created by the residue conformation.

### 3.4. Dependence of hydration on the residue conformation   

Next, we analyzed how the extent of hydration depends on the conformation of the amino-acid χ_1_ torsion angle, *i.e.* the torsion around the CA—CB bond connecting the main chain and side chain. Table 2 ▸ shows the dependence of the hydration on the χ_1_ torsion angle classified as *gauche*+, *gauche*− or *trans*; more detailed information is summarized in Supplementary Table S1. Hydration differs between χ_1_ torsion conformers quite substantially; the differences within the same secondary structure are up to 0.9 waters per amino acid in the case of Thr_E_g^−^ *versus* Thr_E_t. Other large differences involve Asn_E_g^+^ *versus* Asn_E_g^−^ (a difference of 0.5), Asn_T_g^+^ *versus* Asn_T_g^−^ (a difference of 0.6) and analogously Asp_E_g^+^ *versus* Asp_E_g^−^ (a difference of 0.4) and Asp_T_g^+^ *versus* Asp_T_g^−^ (a difference of 0.5). The relative differences are most pronounced for hydrophobic residues, since they are the least hydrated. For instance, the hydration of hydrophobic residues in H_g^+^ conformations is 2.0 times higher than in H_t conformations. Significant differences between the hydration of g^+^, g^−^ and t conformers of χ_1_ were also observed in extended β-sheet conformations, where the most hydrophobic residues are more hydrated in the E_g^−^ and E_t conformations than in E_g^+^ conformations. A possible structural explanation for these differences is discussed below for selected conformers.

### 3.5. Hydration sites of selected conformers   

In order to be able to analyze the spatial distributions of water molecules around amino-acid residues, these needed to be classified beyond their χ_1_ rotameric state. To this end, we performed conformational clustering of amino-acid residues. For each χ_1_ rotameric state and the secondary-structure type of each amino-acid residue (conformational category) we identified the most populated cluster, denoted ‘Conformer1’. The clustering could not be performed for residues with ‘turn’ secondary structure because turns have a large variability in the backbone torsion angles φ and ψ. The percentage of amino-acid residues in each Conformer1 cluster is listed in Supplementary Table S2. For most amino-acid residues Conformer1 represents a substantial fraction of amino-acid residues of a particular χ_1_ rotamer. In fact, it represents 20% or more observations for all residues except Arg, and for more than half of the categories it was even more representative, comprising over 50% of the observations. As expected, this was the case for residues with only one side-chain torsion angle (Cys, Val, Thr and Ser) as well as for Gly, Ala and Pro. The opposite extreme is represented by Arg, which has five side-chain torsion angles, and the size of its Conformer1 cluster ranged from 3.8 to 10.3% depending on the conformational category.

**Figure 2:**
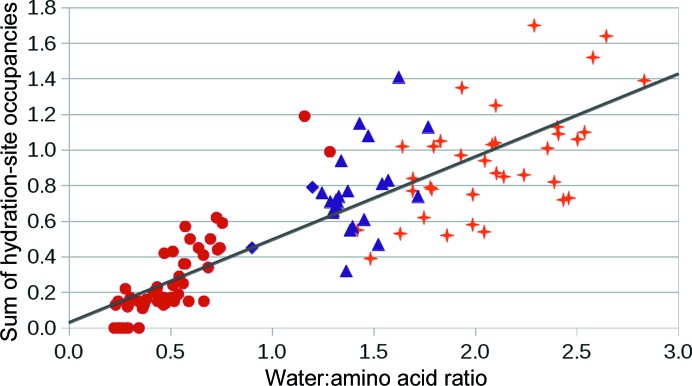
Dependence of the sum of hydration-site occupancies on the total water:amino acid ratio in Conformer1 of 20 amino acids. Data points for hydrophobic residues are marked with circles, moderately polar residues with triangles and very polar residues with crosses. The coefficient of determination *R* ^2^ of the least-squares regression is 0.73 and the slope of the line is 0.47.

Water distributions for all Conformer1 rotamers, *i.e.* 20 amino-acid residues in three χ_1_ rotameric states and two secondary-structure types [exactly two Ala + two Gly + four Pro + (17 × 3 × 2) remaining amino-acid water distributions] were Fourier averaged as described in Schneider *et al.* (1993 ▸). The resulting maxima in the pseudo-electron density represent the preferred positions of water molecules, *i.e.* the hydration sites. Their positions and pseudo-*B* factors were then refined and their pseudo-occupancy was estimated from the pseudo-electron-density value at the position after refinement as described in Schneider & Berman (1995 ▸); the hydration-site positions remained almost the same during refinement. We should note here that since the hydration sites represent a superposition of states, the pseudo-occupancies represent the probability of water being present at the given hydration site; it can also be understood as a measure of the depth of the local minimum of the free-energy hypersurface. The existence of well defined localized hydration sites in virtually all amino acids is in this context a fundamental feature of the water distributions. The sum of occupancies of hydration sites identified by Fourier averaging in a given Conformer1 correlates with the water:amino-acid ratio (Fig. 2 ▸), and encompasses about half of the water that is ordered and observed in the crystal structures. Hydration sites therefore represent a significant portion of the seemingly chaotic distribution of the first hydration shell water. The graph in Fig. 2 ▸ also illustrates the difference in the hydration of hydrophobic and polar residues, the former having a water:amino acid ratio below 0.8 and the latter having a water:amino acid ratio above 1.2. The exceptions are Trp_H_g^+^ and Trp_E_g^−^, with unusually high water:amino acid ratios of 1.2 and 1.3, respectively, and Thr_E_t, with an exceptionally low water:amino acid ratio of 0.9. A possible structural explanation of these observations is discussed below. The coordinates of the hydrated Conformer1 in each category are available in the Supporting Information in PDB file format; the water:amino acid ratios in the Conformer1 clusters are summarized in Supplementary Table S3.

The distances of several hydration sites, *e.g.* those interacting with the side-chain carboxyl group of Asp, were significantly shorter, 2.4–2.6 Å, than would be expected for a typical hydrogen-bond distance of ∼2.8 Å (see Fig. 1 ▸ and Supplementary Tables S4–S10). This can be attributed to strong, negative charge-assisted hydrogen bonds (Gilli & Gilli, 2000 ▸) owing to the deprotonation of a majority of the Asp residues in protein structures. Nevertheless, it cannot be completely ruled out that the shorter distances are also caused to some extent by the presence of lighter cations (typically sodium or magnesium) interacting with the negatively charged carboxylate. These cations have shorter interaction distances than, but similar electron densities to, water oxygen and are often hard to distinguish from water during the refinement process of the diffraction data. This is especially the case for alkali-metal ions such as sodium and potassium, because their coordination spheres are not as regular as those of alkaline-earth metal ions (Zheng *et al.*, 2008 ▸). Therefore, instead of ‘hydration site’ it would be more precise to use the term ‘solvation site’ as the site of all small solvent species, both water and ions.

**Figure 3:**
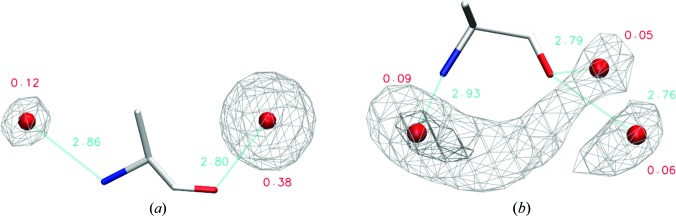
Hydration sites of Ala conformers of (*a*) α-helical and (*b*) extended β-sheet secondary structure. Positions of hydration sites are shown as spheres and their occupancies and distances to the nearest polar atoms are labelled. In the α-helical conformation the water distribution is contoured at an occupancy level of 0.10 waters per amino acid using a mesh. In the β-sheet conformation it is contoured at occupancy levels of 0.04 and 0.08.

**Figure 4:**
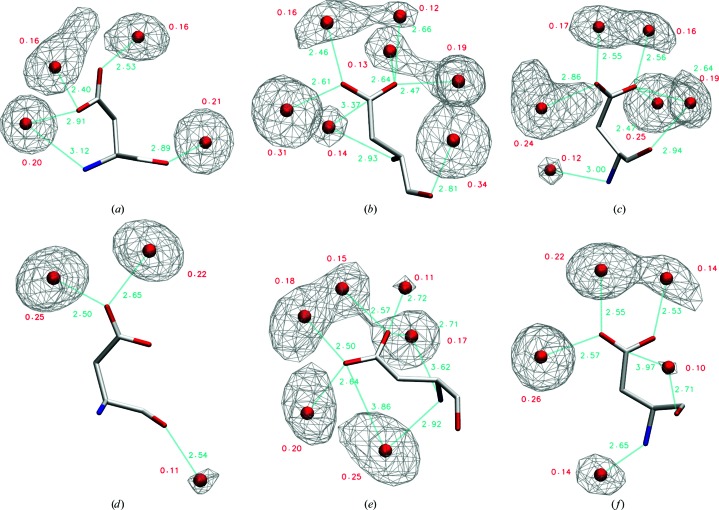
Hydration sites of Asp conformers: (*a*) Asp_H_g^+^, (*b*) Asp_H_g^−^, (*c*) Asp_H_t, (*d*) Asp_E_g^+^, (*e*) Asp_E_g^−^ and (*f*) Asp_E_t. Positions of hydration sites are shown as spheres and their occupancies and distances to the nearest polar atoms are labelled, as well as additional contacts such as the OH–π interaction with the carboxyl group (Asp_H_g^−^ andn Asp_E_t) and bridges between side chain and main chain (Asp_H_g^+^, Asp_H_t and Asp_E_g^−^). Water distributions are contoured at an occupancy level of 0.10 using a mesh.

Further, we discuss the most instructive examples of conformational dependence of amino-acid hydration. Based on the physicochemical nature of the residue and the size of its Conformer1 clusters, we selected Asp, His, Thr, Trp and Tyr residues in α-helical and β-sheet conformations for this detailed analysis. In addition, we discuss the hydration of alanine as a model for the hydration of protein main chain and of leucine as a typical hydrophobic aliphatic amino acid. The water density distributions around the most populated conformers of the selected residues and the corresponding hydration sites are shown in Figs. 3 ▸ and 4 ▸ and Supplementary Figs. S2–S6. The geometric features of the hydration sites of all conformers analyzed in detail are listed in Supplementary Tables S4–S10. The extent of hydration observed for the Conformer1 clusters (see Supplementary Table S3) is similar to that observed for the χ_1_ categories (see Table 2 ▸), showing that Conformer1 is representative of the hydration of the given category. The conformational clustering thus enables the possible structural reasons behind the different hydration levels of different amino-acid conformers to be explored, as discussed below.

#### 3.5.1. Hydration of Ala (Fig. 3 ▸ and Supplementary Table S4)   

The α-helical conformation exhibits an ordered hydration structure, with the main-chain nitrogen and carbonyl each having one localized hydration site. The carbonyl site is very strong, with an occupancy of 0.38. The hydration structure of the β-sheet conformation is completely different: the water distribution is delocalized between the two closely positioned main-chain polar groups and all hydration sites are weak, the strongest being the hydration site of nitrogen, with an occupancy of 0.09 and an elongated shape. The water distribution around the carbonyl O atom creates a partially disordered ring-shaped structure with two weak hydration sites. This conformational dependence of the carbonyl hydration, together with the specific side-chain interactions discussed for the other residues below, may explain the complicated distributions of water molecules around the peptide bond observed in previous studies (Matsuoka & Nakasako, 2009 ▸).

#### 3.5.2. Hydration of Asp (Fig. 4 ▸ and Supplementary Table S5)   

The hydration sites of main-chain nitrogen in both α-helical and β-sheet conformers have similar geometric parameters to those observed in the corresponding Ala conformers. Their occupancies, however, differ between conformers: while in Asp_E_g^+^ the hydration site of nitrogen is of low occupancy, in Asp_E_g^−^ it has an occupancy of 0.25, likely owing to the geometric position of the side-chain OD2 atom, which enables the formation of simultaneous hydrogen bonds to both atoms. Similarly, the N and OD1 atoms share one strong hydration site in Asp_H_g^+^. In Asp_H_g^−^, water in the main-chain nitrogen hydration site can interact with the carboxyl group of the side chain *via* an OH–π interaction.

The hydration sites of the main-chain carbonyl have similar geometries and occupancies to the corresponding Ala conformers. Occupancies are high (∼0.20 or more) in all three helical conformations; in Asp_H_t O shares the hydration site with the side-chain OD1 atom. In contrast, O hydration is weaker (∼0.10 or lower) in extended β-sheet conformations. In Asp_E_t, water in the carbonyl hydration site can interact with the carboxyl group of the side chain *via* an OH–π interaction.

The Asp side-chain OD1 and OD2 atoms are hydrated unequally. In all considered conformers except Asp_H_g^+^ the OD2 atom has two hydration sites, both located in the plane of the carboxyl group, consistent with the Asp hydration sites that have been reported in previous studies (Roe & Teeter, 1993 ▸; Matsuoka & Nakasako, 2009 ▸). No hydration site of OD2 interacts with the main chain. In contrast, the OD1 atom can have also hydration sites located out of the plane of the carboxyl group, which often, but not always, interact with the main-chain polar atoms. These off-plane hydration sites are important, having occupancies of up to 0.20, yet have not been observed in previous studies (Roe & Teeter, 1993 ▸; Matsuoka & Nakasako, 2009 ▸). The greater number and occupancy of hydration sites in the Asp_H_g^−^ and Asp_E_g^−^ conformers correspond to their overall higher hydration compared with the other conformers (see Table 2 ▸).

#### 3.5.3. Hydration of His (Supplementary Fig. S2 and Supplementary Table S6)   

Hydration sites of the main-chain N atom were only observed in the extended β-sheet His conformers. Their geometric parameters are similar to those of the β-sheet conformer of Ala, except for the hydration site in His_E_g^+^, which has a different position, probably owing to the additional off-plane interaction with the ND1 atom. Hydration sites of the main-chain carbonyl were observed in α-helical His conformers, as well as in His_E_t, where the hydration site is stabilized by an additional, very short (3.38 Å) carbon–donor hydrogen bond to CD2. A similar carbon–donor hydrogen bond to the CD2 atom also stabilizes one of the two main-chain carbonyl hydration sites in His_H_g^+^. The other main-chain carbonyl hydration site in His_H_g^+^, which is the stronger site, is in a position similar to that observed in Ala_H and is not stabilized by any bridging interaction. In His_H_g^−^ the carbonyl hydration site also interacts with the side-chain ND1 atom.

Two strong side-chain hydration sites were observed in all six considered histidine conformers. Both the ND1 and NE2 hydration sites are strong (occupancy ∼0.30–0.35); the ND2 sites are usually stronger. The hydrogen-bonding distances of the ND1 sites in His_E_g^+^ and His_H_t are very short at 2.52 Å. It is interesting to note that in both these conformers the ND1 atom also interacts with a main-chain hydration site *via* an off-plane interaction as described by Stollar *et al.* (2004 ▸).

#### 3.5.4. Hydration of Leu (Fig. S3 and Supplementary Table S7)   

Hydration of the main-chain nitrogen was observed in three out of six conformers (two β-sheet and one α-helical); the hydration-site positions are similar to those in the corresponding alanine conformers. Hydration of the main-chain carbonyl was observed only in two α-helical conformers; the hydration-site positions are consistent with those of alanine but their occupancy is variable, from 0.43 in Leu_H_g^+^ to below 0.10 in Leu_H_t. It is interesting to note how the stereochemistry and occupancy of the hydration site correspond to the side-chain rotameric state: strong hydration sites are observed in those rotamers in which the hydrophobic side chain is distant from the hydration site (Leu_H_g^+^; Supplementary Fig. S3*a*), while the hydration-site occupancy is lower (Leu_H_g^−^; Supplementary Fig. S3*b*) or the hydration site is completely absent (Leu_H_t; Supplementary Fig. S3*c*) in rotamers with a short distance between the hydration-site position and the side chain.

#### 3.5.5. Hydration of Thr (Supplementary Fig. S4 and Supplementary Table S8)   

Hydration sites of the main-chain nitrogen were only observed in Thr_H_t and Thr_E_g^−^, both close to the positions of hydration sites in the corresponding alanine conformers. Hydration sites of the main-chain carbonyl group were observed in all helical conformers and for β-sheet conformers only in Thr_E_g^−^ ; the positions of all of these hydration sites are similar to those observed for alanine. The hydration site of the Thr_H_t carbonyl is shared with the side-chain OG1 atom.

The side-chain OG1 atom is typically surrounded by two to three hydration sites with CA—CB—OG1—W torsion angles of about ±90 and 180°; the hydration sites at ±90° often interact with the main chain, *e.g.* the very strong hydration site in Thr_E_g^−^ with an occupancy of 0.45 is in the proximity (3.45 Å) of the main-chain nitrogen, or more specifically in an OH–π interaction with the peptide bond of the preceding residue. In the Thr_H_g^−^ and Thr_H_t Conformer1 cluster hydration sites at around 0° were observed, both stabilized by an additional contact with the main chain. Thus, in almost all analyzed conformers there was at least one hydration site bridging the side chain and main chain.

It is interesting to note that the Thr_E_g^−^ conformer, in which both main-chain hydration sites were resolved together with a very strong side-chain hydration site, also has a higher water:amino acid ratio than the other Thr conformers (see Table 2 ▸ and Supplementary Table S3). In contrast, in the Thr_E_t conformer the hydrophobic nature of the side-chain methyl group, which is close to both main-chain polar groups, may lead to a lower propensity of water to interact with the main chain, resulting in the unusually low hydration in Thr_E_t.

#### 3.5.6. Hydration of Trp (Supplementary Fig. S5 and Supplementary Table S9)   

Hydration of the main chain is variable. The nitrogen hydration site was observed in four out of six conformers, in positions consistent with those observed in the corresponding alanine conformers. In two cases, Trp_E_g^−^ and Trp_H_g^+^, in which the nitrogen hydration site can also interact with the side-chain CD1 atom *via* a carbon–donor hydrogen bond, the hydration sites have very high occupancy (0.45 and 0.53, respectively). These high-occupancy hydration sites explain the unusually high water:amino acid ratio observed for these two Trp conformers (see Table 2 ▸). The hydration site of the main-chain carbonyl was observed in two α-helical conformers, Trp_H_g^+^ and Trp_H_t, both in positions similar to the alanine carbonyl hydration site and both with an additional interaction with the side chain. In the case of Trp_H_t, the carbonyl hydration site interacts with the side-chain NE1 atom *via* an off-plane inter­action (Stollar *et al.*, 2004 ▸); in the case of Trp_H_g^+^ it interacts *via* a carbon-donor hydrogen bond (Petrella & Karplus, 2004 ▸) with the CE3 atom (despite the relatively long distance of 4.5 Å, the hydration site lies precisely in plane with the side chain).

In contrast to the variable main-chain hydration, hydration of the Trp side-chain NE1 atom is very similar in all analyzed Conformer1 clusters both in terms of occupancy (between 0.23 and 0.36) and geometric parameters.

#### 3.5.7. Hydration of Tyr (Supplementary Fig. S6 and Supplementary Table S10)   

The hydration sites of the main-chain N atom were resolved in all conformers in positions generally consistent with those in the corresponding Ala conformers; exceptions are Tyr_H_g^−^ with no hydration site and Tyr_E_g^+^, where the hydration site is deflected by sterical repulsion of the side chain. The water in this hydration site can interact with the phenyl ring *via* an OH–π interaction. A very close contact (carbon–donor hydrogen bond) was observed in Tyr_H_g^+^ between the nitrogen hydration site and the CD2 atom of the side chain.

Main-chain carbonyl hydration sites were resolved in all helical Tyr conformers, but only in one β-sheet conformer, Tyr_E_t. Their positions are similar to those in the corresponding Ala conformers except for Tyr_H_g^+^, in which the hydration site lies exactly in the phenyl-ring plane, with its position stabilized by a carbon-donor hydrogen bond. All of the observed carbonyl hydration sites are in contact with the side chain, either *via* a carbon-donor hydrogen bond or *via* an OH–π interaction.

In all six analyzed tyrosine conformers the side-chain OH atom is hydrated by two hydration sites, both lying in the phenyl-ring plane. Interestingly, the two sites are not completely symmetrical; the hydration site with a W—OH—CZ—CE1 torsion angle around 0° in most cases has a shorter interaction distance than the hydration site at 180° and a slightly lower occupancy (average of 0.26 *versus* 0.24).

## 4. Discussion   

In the present study, we investigated how the number and the stereochemistry of crystallographically ordered waters depend on the amino-acid conformation and environment. Our hypothesis was that the different residue conformers have specific hydration patterns caused by water molecules interacting simultaneously with more than one functional group of the residue. Therefore, we expected that the residue secondary structure and side-chain rotameric state influence the preferred positions of hydration sites. We were also interested in the dependence of hydration on the residue solvent accessibility. We selected a set of 2818 well resolved monomeric protein structures from the PDB and calculated the distance distribution of water molecules around all 20 standard amino-acid residues. The first maximum of distribution lies between 2.80 and 2.95 Å (see Fig. 1 and Supplementary Fig. S1). The majority of these water molecules can be attributed to hydrogen-bonding interactions with the main-chain and side-chain polar protein atoms. The second peak, which appears at ∼3.8 Å, corresponds to a typical van der Waals distance. The two peaks are separated by a minimum located around 3.2–3.3 Å for all residues. Therefore, a value of 3.2 Å was selected as a cutoff distance for all subsequent analyses.

We observed a surprisingly small dependence of the hydration of a residue on its SASA. The water:amino acid ratios in residues with SASAs of >30% and residues with a SASA of between 5 and 30% are similar (see Table 1 ▸). Moreover, in highly polar residues a significant portion of hydration was also retained at low SASA values of below 5%. This shows that the water molecules are to a large extent inseparable from these very polar residues, as the free-energy penalty for complete desolvation would be too high.

The extent of hydration and its stereochemistry depends on the secondary structure in which an amino-acid residue occurs. Moreover, different trends were observed for the extent of hydration of polar and of hydrophobic residues in α-helical, extended β-sheet and turn secondary structures (see Table 1 ▸). For polar residue only small differences were observed between the water:amino acid ratios for residues in different secondary structures, while for hydrophobic residues the turn secondary structures are about twice as hydrated as both α-helices and β-sheets. This again points to the fact that the hydration of polar residues is a characteristic feature of the residue itself, whereas the hydration of hydrophobic residues is dependent on the structural context.

The hydration also depends on the conformation of the residue, specifically on the rotameric state of its χ_1_ torsion angle (see Table 2 ▸). We observed significant differences in the extent of hydration between χ_1_ torsion conformers of up to 0.9 waters per amino acid in the case of Thr_E_g^−^ *versus* Thr_E_t; significant differences in the hydration of χ_1_ conformers were also observed for Phe, Asn and Asp residues.

To inspect the spatial distribution of water molecules around amino-acid residues and to structurally interpret the observed differences, the residues have to be conformationally clustered beyond their χ_1_ torsion angle. To this end, the residues were clustered using the QT algorithm with the r.m.s.d. as the distance function in categories defined by the residue, its secondary structure and the χ_1_ angle. In the largest conformational cluster within each category (see Supplementary Table S2), the water distribution was obtained using Fourier averaging (Schneider *et al.*, 1993 ▸). The hydration sites were then identified as maxima in the density distribution; the coordinates and occupancies are available in the Supporting Information in PDB file format.

The results for seven representative amino-acid residues were analyzed in detail (see Figs. 3 ▸ and 4 ▸, Supplementary Figs. S2–S6 and Supplementary Tables S3–S10). They show well localized isolated hydration sites but also frequent occurrences of hydration sites shared by main-chain and side-chain protein atoms. We also observed different hydration-site positions and occupancies for different conformers of the same residue type, and have described several occurrences of hydration sites with less canonical contacts, such as carbon-donor hydrogen bonds, OH–π interactions and off-plane interactions with aromatic heteroatoms. The conformational dependence and the role of the specific side-chain interactions stabilizing the main-chain hydration can also explain the previously observed overlapping distributions of water molecules around peptide bonds (Matsuoka & Nakasako, 2009 ▸).

The limitation of our approach is the growing number of conformational states into which residues with longer side chains can be subdivided. For residues with shorter side chains, the largest cluster represents the majority of the residues. In such cases, the six categories used here are sufficient to describe the conformational diversity of residues in α-helical and extended β-sheet secondary structures. However, with longer side chains and higher numbers of possible torsion-angle combinations, the percentage of conformers represented by the largest cluster decreases rapidly. An extreme example is arginine, with five side-chain torsion angles, where the size of the largest cluster in the six conformational categories ranges between 4 and 10%. On the other hand, the larger distance between the side-chain and main-chain polar atoms in residues such as arginine and lysine leads to a lesser mutual influence between their hydration sites. Therefore, an independent description of the main-chain and side-chain hydration, as performed in other studies (Matsuoka & Nakasako, 2009 ▸), becomes more justified for such residues.

The necessity to cluster residues by their conformation has also prevented us from applying this approach to residues classified as turn secondary structure, because the backbone torsion angles are very variable. This is of course a major drawback, since this type of secondary structure is the most hydrated, as shown by our own statistical analysis (see Table 1 ▸). It is nevertheless possible to envision different clustering schemes in which several sequentially adjacent residues would be taken into account and clustered based on their overall conformational class, such as the peptide block (Joseph *et al.*, 2010 ▸). Such an approach would enable a distinction to be made between different types of turns and thus allow their specific hydration patterns to be resolved.

The challenge lying ahead is thus to combine different clustering approaches and to develop a dependable energy function that would realistically and reliably describe the interaction between protein and water of hydration in any geometric configuration, such as the elastic potential build for fitting proteins or drugs into DNA helices (Ge *et al.*, 2005 ▸). Empirical studies contribute to the development of computational approaches in structural bioinformatics areas such as protein structure prediction (Papoian *et al.*, 2004 ▸; Jiang *et al.*, 2005 ▸), prediction of protein interactions with ligands (De Beer *et al.*, 2010 ▸) and with other biomolecules (Bueno *et al.*, 2010 ▸), and protein–protein docking (Kastritis *et al.*, 2013 ▸; Parikh & Kellogg, 2014 ▸). We suggest that the conformation-specific hydration of amino-acid residues in proteins could also be used for more accurate water placement in crystallographic structure refinement and validation (Matsuoka & Nakasako, 2013 ▸) and to predict the positions of ordered water molecules around proteins in the preparation of MD simulations (Wallnoefer *et al.*, 2011 ▸). The empirically determined hydration sites may also help to understand the role of the large numbers of well ordered water molecules with extremely small thermal vibrations observed at protein–protein or protein–DNA interfaces (Schneider *et al.*, 2014 ▸).

To conclude, we believe that our study represents the most complex model of protein hydration to date, taking into account dependence on secondary structure and side-chain conformation. We have shown that different conformers of the same amino-acid residue can have strikingly different hydration patterns owing to the fact that water molecules often interact with more than one functional group and that the hydration pattern also depends on the secondary structure in which the amino acid occurs.

## Supplementary Material

Supplementary tables and figures.. DOI: 10.1107/S1399004715015679/dw5143sup1.pdf

Zip archive with the structures of 110 hydrated amino-acid residue conformers in PDB file format.. DOI: 10.1107/S1399004715015679/dw5143sup2.zip

## Figures and Tables

**Table 1:** Dependence of the water:amino acid ratio on residue SASA and residue secondary structure Residues which are discussed in greater detail in the text are highlighted in bold.

		SASA			Secondary structure†		
		5%	530%	>30%	H	E	T
Hydrophobic‡	**Ala**	**0.4**	**1.0**	**1.3**	**0.6**	**0.4**	**1.1**
	**Leu**	**0.4**	**0.8**	**1.1**	**0.4**	**0.5**	**1.0**
	**Trp**	**0.7**	**1.2**	**1.5**	**0.7**	**0.9**	**1.4**
	All	0.4	0.9	1.2	0.4	0.5	1.0
Moderately polar‡	**His**	**1.1**	**1.6**	**1.9**	**1.5**	**1.3**	**1.7**
	Ser	1.0	1.6	2.0	1.5	1.4	1.7
	**Thr**	**1.0**	**1.6**	**2.0**	**1.3**	**1.5**	**1.6**
	**Tyr**	**1.0**	**1.6**	**1.9**	**1.2**	**1.3**	**1.8**
	All	1.0	1.6	2.0	1.3	1.4	1.7
Very polar‡	Arg	1.5	2.1	2.3	2.1	2.0	2.4
	Asn	1.3	2.2	2.4	2.1	1.9	2.2
	**Asp**	**1.7**	**2.6**	**2.7**	**2.7**	**2.3**	**2.5**
	Gln	1.3	2.2	2.3	2.0	2.0	2.3
	Glu	1.7	2.5	2.5	2.3	2.4	2.6
	Lys	1.3	1.8	1.9	1.7	1.7	2.0
	All	1.5	2.3	2.3	2.2	2.0	2.4
All amino acids		0.6	1.5	2.0	1.2	1.0	1.6

† H, -helix; E, extended -sheet; T, turn.

‡ Definition of residue type according to Rose *et al.* (1985 ▸).

**Table 2:** Dependence of the water:amino acid ratio on _1_ torsion-angle conformation (g^+^/g/t) in various secondary structures Residues which are discussed in greater detail in the text are highlighted in bold.

	-Helix (H)			-Sheet (E)			Turn (T)		
	g^+^	g	t	g^+^	g	t	g^+^	g	t
Hydrophobic†									
**Leu**	**0.6**	**0.4**	**0.2**	**0.2**	**0.5**	**0.5**	**1.0**	**1.0**	**0.9**
**Trp**	**1.0**	**0.7**	**0.7**	**0.8**	**1.0**	**0.8**	**1.3**	**1.4**	**1.4**
All‡	0.6	0.4	0.3	0.4	0.5	0.5	0.8	0.9	1.0
Moderately polar†									
**His**	**1.7**	**1.5**	**1.4**	**1.2**	**1.3**	**1.3**	**1.6**	**1.8**	**1.6**
Ser	1.5	1.4	1.5	1.4	1.6	1.3	1.6	1.8	1.5
**Thr**	**1.3**	**1.3**	**1.3**	**1.4**	**1.8**	**0.9**	**1.6**	**1.7**	**1.4**
**Tyr**	**1.7**	**1.2**	**1.2**	**1.2**	**1.3**	**1.3**	**1.7**	**1.7**	**1.9**
All	1.4	1.3	1.3	1.3	1.5	1.2	1.6	1.8	1.6
Very polar†									
Arg	2.4	2.2	1.9	1.8	2.0	2.0	2.4	2.4	2.4
Asn	1.8	2.2	2.0	1.5	2.0	1.7	1.8	2.4	2.2
**Asp**	**2.3**	**2.8**	**2.4**	**2.1**	**2.5**	**2.2**	**2.3**	**2.8**	**2.4**
Gln	2.0	2.0	1.9	1.8	2.0	2.0	2.0	2.3	2.3
Glu	2.3	2.3	2.3	2.1	2.4	2.4	2.5	2.6	2.7
Lys	2.0	1.8	1.6	1.5	1.7	1.8	2.0	2.0	2.1
All	2.2	2.3	2.0	1.8	2.1	2.0	2.2	2.4	2.3
All amino acids‡	1.4	1.4	1.1	1.1	1.1	1.1	1.5	1.8	1.8

† Definition of residue type according to Rose *et al.* (1985 ▸).

‡ Gly and Ala residues are not included.
