# Supplementary material for: Structure of the ordered hydration of amino acids in proteins: analysis of crystal structures
Source: Acta Crystallogr D Biol Crystallogr. 2015 Oct 27;71(Pt 11):2192–202. doi: 10.1107/S1399004715015679 (PMC4631476; doi:10.1107/S1399004715015679)
Supplement: Supplementary file 1 [file d-71-02192-sup1.pdf]

Supporting information

To the manuscript

Structure of the Ordered Hydration of Amino Acids in Proteins: Analysis of Crystal Structures

Lada Biedermannová* and Bohdan Schneider

Laboratory of Biomolecular Recognition, Institute of Biotechnology,

Academy of Sciences of the Czech Republic, CZ-142 20 Prague, Czech Republic

SUPPLEMENTARY TABLES

- TABLE S1. Number of AA residues in categories defined by residue type, secondary structure (H/E/T) and χ1 rotameric state (g+/g-/t).
- TABLE S2. Conformer1 cluster size as percentage of AA residues of the given category defined by residue type, secondary structure (H/E) and χ1 rotameric state (g+/g-/t).
- TABLE S3. Water/AA ratio in Conformer1 clusters in categories defined by residue type, secondary structure (H/E) and χ1 torsion angle conformation (g+/g-/t).
- TABLE S4. Characteristics of Ala hydration sites.
- TABLE S5. Characteristics of Asp hydration sites.
- TABLE S6. Characteristics of His hydration sites.
- TABLE S7. Characteristics of Leu hydration sites.
- TABLE S8. Characteristics of Thr hydration sites.
- TABLE S9. Characteristics of Trp hydration sites.
- TABLE S10. Characteristics of Tyr hydration sites.

SUPPLEMENTARY FIGURES

- FIGURE S1. Distance distribution of waters around A) polar residues B) hydrophobic residues.
- FIGURE S2. Hydration sites of His conformers.
- FIGURE S3. Hydration sites of Leu conformers.
- FIGURE S4. Hydration sites of Thr conformers.
- FIGURE S5. Hydration sites of Trp conformers.
- FIGURE S6. Hydration sites of Tyr conformers.

SUPPLEMENTARY FILES

- Zip archive with the structures of 110 most populated conformers of all 20 AA residues with their hydration sites in PDB file format.

Table S1 Number of AA residues in categories defined by residue type, secondary structure (H/E/T) and χ1 rotameric state (g+/g-/t).

		α-helix (H)			β-sheet (E)			Turn (T)		
		g+	g-	t	g+	g-	t	g+	g-	t
Hydrophobic	Cys	98	1453	444	512	1082	766	377	827	458
	Ile	553	9153	768	1757	10854	1187	862	1876	415
	**Leu**	**55**	**13753**	**7243**	**409**	**8003**	**6299**	**75**	**4977**	**1279**
	Met	71	2524	1022	286	1208	893	107	760	238
	Phe	101	3024	4106	1749	4783	1810	349	2440	785
	Pro	977	2728	0	1466	1237	0	5533	4493	0
	**Trp**	**186**	**1128**	**1448**	**526**	**1741**	**687**	**330**	**770**	**399**
	Val	545	919	9727	1522	3397	14047	435	1407	2570
Mod. polar	**His**	**158**	**1916**	**1553**	**547**	**1603**	**1175**	**486**	**1870**	**901**
	Ser	3318	3354	1872	2962	2013	2473	4744	2415	1905
	**Thr**	**2187**	**5432**	**88**	**3506**	**5086**	**1308**	**4661**	**1633**	**429**
	**Tyr**	**149**	**2564**	**3563**	**1426**	**4237**	**1890**	**418**	**2255**	**876**
Very polar	Arg	319	5808	4343	759	3036	2267	605	3138	1043
	Asn	239	4887	1177	468	1980	1696	1675	4636	2676
	**Asp**	**434**	**7132**	**1471**	**492**	**1918**	**2462**	**3164**	**5012**	**3799**
	Gln	202	5598	3302	506	2024	1817	421	2780	797
	Glu	545	9431	5925	733	3087	2726	865	4199	1664
	Lys	326	6453	5067	623	3340	2733	643	4387	1517

Note: Ala and Gly residues are not listed in the table due to undefined χ1 rotameric state (denoted in category name as “NA”). Number of AA residues in Ala_E_NA, Ala_H_NA and Ala_T_NA categories is 8916, 21764 and 8186, respectively. Number of AA residues in Gly_E_NA, Gly_H_NA and Gly_T_NA categories is 7346, 5952 and 16912, respectively.

Table S2 *Conformer1* cluster size as percentage of AA residues of the given category defined by residue type, secondary structure (H/E) and χ1 rotamer (g+/g-/t).

		α-helix (H)			β-sheet (E)		
		g+	g-	t	g+	g-	t
Hydrophobic	Cys	97	99	100	95	97	98
	Ile	94	81	59	81	73	87
	**Leu**	**82**	**94**	**90**	**60**	**76**	**85**
	Met	32	25	25	28	20	25
	Phe	35	30	59	45	40	50
	Pro	100	100	N/A*	95	97	N/A*
	**Trp**	**51**	**24**	**23**	**24**	**28**	**13**
	Val	99	99	100	98	88	98
Mod. polar	**His**	**44**	**34**	**50**	**36**	**43**	**28**
	Ser	100	100	99	94	96	97
	**Thr**	**100**	**100**	**97**	**91**	**99**	**99**
	**Tyr**	**28**	**25**	**44**	**37**	**32**	**40**
Very polar	Arg	7	10	4	10	5	5
	Asn	56	75	56	53	52	41
	**Asp**	**66**	**88**	**71**	**47**	**65**	**57**
	Gln	19	33	28	25	20	27
	Glu	24	40	34	37	36	41
	Lys	37	33	28	43	24	30

Note: Ala and Gly residues are not listed in the table due to undefined χ1 rotameric state (denoted in category name as “NA”). *Conformer1* cluster size in categories Ala_E_NA and Ala_H_NA is 96% and 100%, respectively. *Conformer1* cluster size in categories Gly_E_NA and Gly_H_NA is 95% and 99%, respectively.
*Categories Pro_E_t and Pro_H_t contain no AA residues due to steric restrictions.

Table S3 Water/AA ratio in *Conformer1* clusters in categories defined by residue type, secondary structure (H/E) and $\chi 1$ rotamer (g+/g-/t).

		α-helix (H)			β-sheet (E)		
		g+	g-	t	g+	g-	t
Hydrophobic	Cys	0.7	0.3	0.3	0.3	0.5	0.5
	Ile	0.7	0.3	0.5	0.3	0.4	0.4
	**Leu**	**0.5**	**0.4**	**0.2**	**0.3**	**0.5**	**0.5**
	Met	0.6	0.4	0.2	0.2	0.3	0.5
	Phe	0.7	0.5	0.3	0.2	0.4	0.6
	Pro	0.5	0.5	N/A*	0.7	0.8	N/A*
	**Trp**	**1.2**	**0.7**	**0.7**	**0.6**	**1.3**	**0.7**
	Val	0.6	0.6	0.3	0.4	0.3	0.5
Mod. polar	**His**	**1.6**	**1.7**	**1.5**	**1.3**	**1.3**	**1.2**
	Ser	1.5	1.4	1.5	1.3	1.5	1.3
	**Thr**	**1.3**	**1.3**	**1.4**	**1.4**	**1.8**	**0.9**
	**Tyr**	**1.6**	**1.4**	**1.2**	**1.3**	**1.3**	**1.4**
Very polar	Arg	2.4	2.6	1.9	1.6	1.5	2.1
	Asn	1.8	2.3	2.0	1.4	2.0	1.7
	**Asp**	**2.5**	**2.8**	**2.4**	**2.0**	**2.5**	**2.2**
	Gln	1.8	2.1	1.7	1.8	2.0	2.1
	Glu	2.5	2.4	2.6	2.1	2.4	2.4
	Lys	2.1	1.9	1.7	1.6	1.8	1.9

Note: Ala and Gly residues are not listed in the table due to undefined $\chi 1$ rotameric state (denoted in category name as "NA"). Water/AA ratio in *Conformer1* clusters Ala_E_NA and Ala_H_NA is 0.4 and 0.6, respectively; in *Conformer1* clusters Gly_E_NA and Gly_H_NA the water/AA ratio is 0.4 and 0.6, respectively.

*Categories Pro_E_t and Pro_H_t contain no AA residues; the water/AA ratio is thus undefined (N/A).

Table S4 Characteristics of Ala hydration sites.

Conformer1	HS	Occup.	H-bond	Dist. (Å)	Angle	Deg.	Torsion angle	Deg.
Ala_E_NA	W1	0.09	W1-N	2.93	W1-N-CA	119	W1-N-CA-C	95
	W2	0.06	W2-O	2.76	W2-O-C	148	W2-O-C-CA	174
	W3	0.05	W3-O	2.79	W3-O-C	115	W3-O-C-CA	-97
Ala_H_NA	W1	0.38	W1-O	2.80	W1-O-C	121	W1-O-C-CA	27
	W2	0.12	W2-N	2.86	W2-N-CA	113	W2-N-CA-C	131

Note: The undefined $\chi1$ rotameric state of Ala is denoted in category name as “NA”.

Table S5 Characteristics of Asp hydration sites

Conformer1	HS	Occup.	H-bond	Dist. (Å)	Angle	Deg.	Torsion angle	Deg.	Bridge	Dist. (Å)	Note
Asp_E_g-	W1	0.25	W1-N	2.92	W1-N-CA	118	W1-N-CA-C	95	W1-OD2	3.86	
	W2	0.20	W2-OD2	2.64	W2-OD2-CG	131	W2-OD2-CG-CB	-3			
	W3	0.18	W3-OD2	2.50	W3-OD2-CG	120	W3-OD2-CG-CB	158			
	W4	0.17	W4-OD1	2.71	W4-OD1-CG	125	W4-OD1-CG-CB	93	W4-N	3.62	*
	W5	0.15	W5-OD1	2.57	W5-OD1-CG	116	W5-OD1-CG-CB	-162			
	W6	0.11	W6-OD1	2.72	W6-OD1-CG	100	W6-OD1-CG-CB	-66			*
Asp_E_g+	W1	0.25	W1-OD2	2.50	W1-OD2-CG	130	W1-OD2-CG-CB	-13			
	W2	0.22	W2-OD2	2.65	W2-OD2-CG	121	W2-OD2-CG-CB	-151			
	W3	0.11	W3-O	2.54	W3-O-C	149	W3-O-C-CA	11			
Asp_E_t	W1	0.26	W1-OD2	2.57	W1-OD2-CG	126	W1-OD2-CG-CB	-4			
	W2	0.22	W2-OD2	2.55	W2-OD2-CG	125	W2-OD2-CG-CB	178			
	W3	0.14	W3-OD1	2.53	W3-OD1-CG	129	W3-OD1-CG-CB	153			
	W4	0.14	W4-N	2.65	W4-N-CA	120	W4-N-CA-C	88			
	W5	0.10	W5-O	2.71	W5-O-C	123	W5-O-C-CA	-104	W5-CG	3.97	†
Asp_H_g-	W1	0.34	W1-O	2.81	W1-O-C	121	W1-O-C-CA	24			
	W2	0.31	W2-OD2	2.61	W2-OD2-CG	128	W2-OD2-CG-CB	-2			
	W3	0.19	W3-OD1	2.47	W3-OD1-CG	141	W3-OD1-CG-CB	-41	W3-N	4.18	*
	W4	0.16	W4-OD2	2.46	W4-OD2-CG	132	W4-OD2-CG-CB	177			
	W5	0.14	W5-N	2.93	W5-N-CA	117	W5-N-CA-C	126	W5-CG	3.37	†
	W6	0.13	W6-OD1	2.64	W6-OD1-CG	122	W6-OD1-CG-CB	122	W6-N	4.26	*
	W7	0.12	W7-OD1	2.66	W7-OD1-CG	118	W7-OD1-CG-CB	-155			
Asp_H_g+	W1	0.21	W1-O	2.89	W1-O-C	126	W1-O-C-CA	27			
	W2	0.20	W2-OD1	2.91	W2-OD1-CG	98	W2-OD1-CG-CB	-71	W2-N	3.12	*‡
	W3	0.16	W3-OD2	2.53	W3-OD2-CG	129	W3-OD2-CG-CB	-12			
	W4	0.16	W4-OD1	2.4	W4-OD1-CG	109	W4-OD1-CG-CB	-175			
Asp_H_t	W1	0.25	W1-OD1	2.47	W1-OD1-CG	123	W1-OD1-CG-CB	20	W1-N	4.27	

W2	0.24	W2-OD2	2.86	W2-OD2-CG	124	W2-OD2-CG-CB	1			
W3	0.19	W3-OD1	2.64	W3-OD1-CG	126	W3-OD1-CG-CB	-82	W3-O	2.94	*‡
W4	0.17	W4-OD2	2.55	W4-OD2-CG	128	W4-OD2-CG-CB	-160			
W5	0.16	W5-OD1	2.56	W5-OD1-CG	118	W5-OD1-CG-CB	169			
W6	0.12	W6-N	3.00	W6-N-CA	113	W6-N-CA-C	130			

*Site out of carboxyl plane, not observed in previous studies

†OH-π interaction

‡ Overlap with main-chain hydration site

Table S6 Characteristics of His hydration sites

Conformer1	HS	Occup.	H-bond	Dist. (Å)	Angle	Deg.	Torsion angle	Deg.	Bridge	Dist. (Å)	Note
His_E_g+	W1	0.35	W1-ND1	2.52	W1-ND1-CG	136	W1-ND1-CG-CB	-10	W1-N	3.59	
	W2	0.28	W2-NE2	2.84	W2-NE2-CD2	118	W2-NE2-CD2-CG	-172	-		
	W3	0.11	W3-N	2.89	W3-N-CA	125	W3-N-CA-C	38	W3-ND1	3.57	*
His_E_g-	W1	0.31	W1-NE2	2.69	W1-NE2-CD2	135	W1-NE2-CD2-CG	-176			
	W2	0.26	W2-ND1	2.84	W2-ND1-CG	119	W2-ND1-CG-CB	-4			
	W3	0.15	W3-N	2.82	W3-N-CA	119	W3-N-CA-C	76	W3-O	3.76	
His_E_t	W1	0.30	W1-NE2	2.73	W1-NE2-CD2	130	W1-NE2-CD2-CG	178			
	W2	0.24	W2-ND1	2.86	W2-ND1-CG	119	W2-ND1-CG-CB	0			
	W3	0.12	W3-O	2.85	W3-O-C	122	W3-O-C-CA	-100	W3-CD2	3.38	†
	W4	0.10	W4-N	2.68	W4-N-CA	120	W4-N-CA-C	98			
His_H_g+	W1	0.55	W1-ND1	2.87	W1-ND1-CG	112	W1-ND1-CG-CB	-18	W1-N	3.2	
	W2	0.44	W2-O	2.68	W2-O-C	122	W2-O-C-CA	20			
	W3	0.29	W3-NE2	2.72	W3-NE2-CD2	139	W3-NE2-CD2-CG	-168			
	W4	0.13	W4-O	2.91	W4-O-C	110	W4-O-C-CA	105	W4-CD2	3.45	†
His_H_g-	W1	0.41	W1-ND1	2.76	W1-ND1-CG	125	W1-ND1-CG-CB	5	W1-O		
	W2	0.33	W2-NE2	2.76	W2-NE2-CD2	127	W2-NE2-CD2-CG	176			
His_H_t	W1	0.25	W1-ND1	2.52	W1-ND1-CG	130	W1-ND1-CG-CB	-14			
	W2	0.20	W2-NE2	2.78	W2-NE2-CD2	119	W2-NE2-CD2-CG	-178			
	W3	0.16	W3-O	2.62	W3-O-C	133	W3-O-C-CA	7	W3-ND1	3.34	*

[*] Off-plane interaction with ring nitrogen

[†] Carbon-donor H-bond

Table S7 Characteristics of Leu hydration sites

Conformer1	HS	Occup.	H-bond	Dist. (Å)	Angle	Deg.	Torsion angle	Deg.
Leu_E_g+	none	N/A	N/A	N/A	N/A	N/A	N/A	N/A
Leu_E_g-	W1	0.14	W1-N	2.89	W1-N-CA	118	W1-N-CA-C	85
Leu_E_t	W1	0.14	W1-N	2.87	W1-N-CA	118	W1-N-CA-C	92
Leu_H_g+	W1	0.43	W1-O	2.89	W1-O-C	127	W1-O-C-CA	13
Leu_H_g-	W1	0.22	W1-O	2.72	W1-O-C	126	W1-O-C-CA	24
Leu_H_t	W1	0.13	W1-N	2.90	W1-N-CA	117	W1-N-CA-C	129

Table S8 Characteristics of Thr hydration sites

Conformer1	HS	Occup.	H-bond	Dist. (Å)	Angle	Deg.	Torsion angle	Deg.	Bridge	Dist. (Å)	Note
Thr_E_g+	W1	0.22	W1-OG1	2.84	W1-OG1-CB	115	W1-OG1-CB-CA	91	W1-O	3.59	
	W2	0.18	W2-OG1	2.50	W2-OG1-CB	117	W2-OG1-CB-CA	-172			
	W3	0.15	W3-OG1	2.80	W3-OG1-CB	112	W3-OG1-CB-CA	-92	W3-N	3.72	
Thr_E_g-	W1	0.45	W1-OG1	2.73	W1-OG1-CB	114	W1-OG1-CB-CA	85	W1-N	3.45	*
	W2	0.30	W2-OG1	2.67	W2-OG1-CB	123	W2-OG1-CB-CA	-157			
	W3	0.23	W3-N	2.85	W3-N-CA	120	W3-N-CA-C	73			
	W4	0.15	W4-O	2.83	W4-O-C	118	W4-O-C-CA	-97			
Thr_E_t	W1	0.16	W1-OG1	2.86	W1-OG1-CB	106	W1-OG1-CB-CA	79	-		
	W2	0.15	W2-OG1	2.48	W2-OG1-CB	135	W2-OG1-CB-CA	-107	-		
	W3	0.14	W3-OG1	2.66	W3-OG1-CB	116	W3-OG1-CB-CA	176	-		
Thr_H_g+	W1	0.30	W1-O	2.72	W1-O-C	128	W1-O-C-CA	8	-		
	W2	0.22	W2-OG1	2.56	W2-OG1-CB	127	W2-OG1-CB-CA	93	W2-O	4.18	
	W3	0.19	W3-OG1	2.70	W3-OG1-CB	117	W3-OG1-CB-CA	-168	-		
Thr_H_g-	W1	0.22	W1-OG1	2.64	W1-OG1-CB	112	W1-OG1-CB-CA	160	-		
	W2	0.20	W2-O	2.74	W2-O-C	127	W2-O-C-CA	12	-		
	W3	0.16	W3-OG1	2.63	W3-OG1-CB	113	W3-OG1-CB-CA	-73	-		
	W4	0.13	W4-OG1	2.74	W4-OG1-CB	137	W4-OG1-CB-CA	5	W4-N	3.63	
Thr_H_t	W1	0.18	W1-N	2.88	W1-N-CA	117	W1-N-CA-C	136			†
	W2	0.14	W2-OG1	2.97	W2-OG1-CB	130	W2-OG1-CB-CA	14	W2-O	3.07	†

*Unusually high occupancy

†Small cluster size (low number of AA)

Table S9 Characteristics of Trp hydration sites

Conformer1	HS	Occup.	H-bond	Dist. (Å)	Angle	Deg.	Torsion angle	Deg.	Bridge	Dist. (Å)	Note
Trp_E_g+	W1	0.36	W1-NE1	3.07	W1-NE1-CD1	118	W1-NE1-CD1-CG	-171	-		
	W2	0.10	W2-O	3.12	W2-O-C	119	W2-O-C-CA	-132	W2-NE1	4.23	*
Trp_E_g-	W1	0.45	W1-N	3.00	W1-N-CA	119	W1-N-CA-C	82	W1-CD1	3.86	†
	W2	0.31	W2-NE1	2.88	W2-NE1-CD1	125	W2-NE1-CD1-CG	-174	-		
	W3	0.12	W3-O	2.63	W3-O-C	146	W3-O-C-CA	64	-		
	W4	0.11	W4-O	2.69	W4-O-C	139	W4-O-C-CA	-57	-		
Trp_E_t	W1	0.23	W1-NE1	2.87	W1-NE1-CD1	137	W1-NE1-CD1-CG	-174	-		
	W2	0.11	W2-N	2.86	W2-N-CA	118	W2-N-CA-C	86	-		
Trp_H_g+	W1	0.53	W1-N	2.84	W1-N-CA	125	W1-N-CA-C	143	W1-CD1	3.37	†
	W2	0.35	W2-O	2.62	W2-O-C	125	W2-O-C-CA	20	W2-CE3	4.47	†
	W3	0.31	W3-NE1	2.83	W3-NE1-CD1	124	W3-NE1-CD1-CG	-180	-		
Trp_H_g-	W1	0.30	W1-NE1	2.86	W1-NE1-CD1	130	W1-NE1-CD1-CG	162	-		
	W2	0.11	W2-N	2.93	W2-N-CA	119	W2-N-CA-C	107	W2-CD1	3.65	†
Trp_H_t	W1	0.30	W1-NE1	2.76	W1-NE1-CD1	132	W1-NE1-CD1-CG	-180			
	W2	0.20	W2-O	2.79	W2-O-C	128	W2-O-C-CA	20	W2-NE1	3.36	*

*Off-plane interaction with ring nitrogen

†Carbon-donor H-bond

Table S10 Characteristics of Tyr hydration sites

Conformer1	HS	Occup.	H-bond	Dist. (Å)	Angle	Deg.	Torsion angle	Deg.	Bridge	Dist. (Å)	Note
Tyr_E_g+	W1	0.30	W1-OH	2.61	W1-OH-CZ	122	W1-OH-CZ-CE1	-173			
	W2	0.29	W2-OH	2.57	W2-OH-CZ	123	W2-OH-CZ-CE1	-1			
	W3	0.10	W3-N	3.01	W3-N-CA	125	W3-N-CA-C	40	W3-CD2	3.77	*
Tyr_E_g-	W1	0.28	W1-OH	2.86	W1-OH-CZ	123	W1-OH-CZ-CE1	179			
	W2	0.25	W2-OH	2.47	W2-OH-CZ	119	W2-OH-CZ-CE1	5			
	W3	0.12	W3-N	3.04	W3-N-CA	121	W3-N-CA-C	73	W3-CD2	4.01	†
Tyr_E_t	W1	0.22	W1-OH	2.58	W1-OH-CZ	121	W1-OH-CZ-CE1	178			
	W2	0.14	W2-OH	2.60	W2-OH-CZ	122	W2-OH-CZ-CE1	19			
	W3	0.11	W3-N	2.95	W3-N-CA	119	W3-N-CA-C	80			
	W4	0.10	W4-O	2.81	W4-O-C	131	W4-O-C-CA	-113	W4-CD2	3.76	†
Tyr_H_g+	W1	0.26	W1-OH	2.80	W1-OH-CZ	116	W1-OH-CZ-CE1	-180			
	W2	0.24	W2-OH	2.67	W2-OH-CZ	116	W2-OH-CZ-CE1	-27			
	W3	0.19	W3-N	2.87	W3-N-CA	124	W3-N-CA-C	150	W3-CD2	3.35	†
	W4	0.14	W4-O	2.40	W4-O-C	137	W4-O-C-CA	103	W4-CD1	4.14	†
Tyr_H_g-	W1	0.31	W1-O	2.64	W1-O-C	122	W1-O-C-CA	3	W1-CD1	3.90	†
	W2	0.24	W2-OH	2.73	W2-OH-CZ	120	W2-OH-CZ-CE1	179			
	W3	0.22	W3-OH	2.44	W3-OH-CZ	118	W3-OH-CZ-CE1	5			
Tyr_H_t	W1	0.27	W1-OH	2.49	W1-OH-CZ	120	W1-OH-CZ-CE1	-3			
	W2	0.25	W2-OH	2.64	W2-OH-CZ	123	W2-OH-CZ-CE1	179			
	W3	0.16	W3-O	2.68	W3-O-C	126	W3-O-C-CA	3	W3-CD1	3.37	*
	W4	0.11	W4-N	2.98	W4-N-CA	115	W4-N-CA-C	134			

*OH-π interaction

†Carbon-donor H-bond

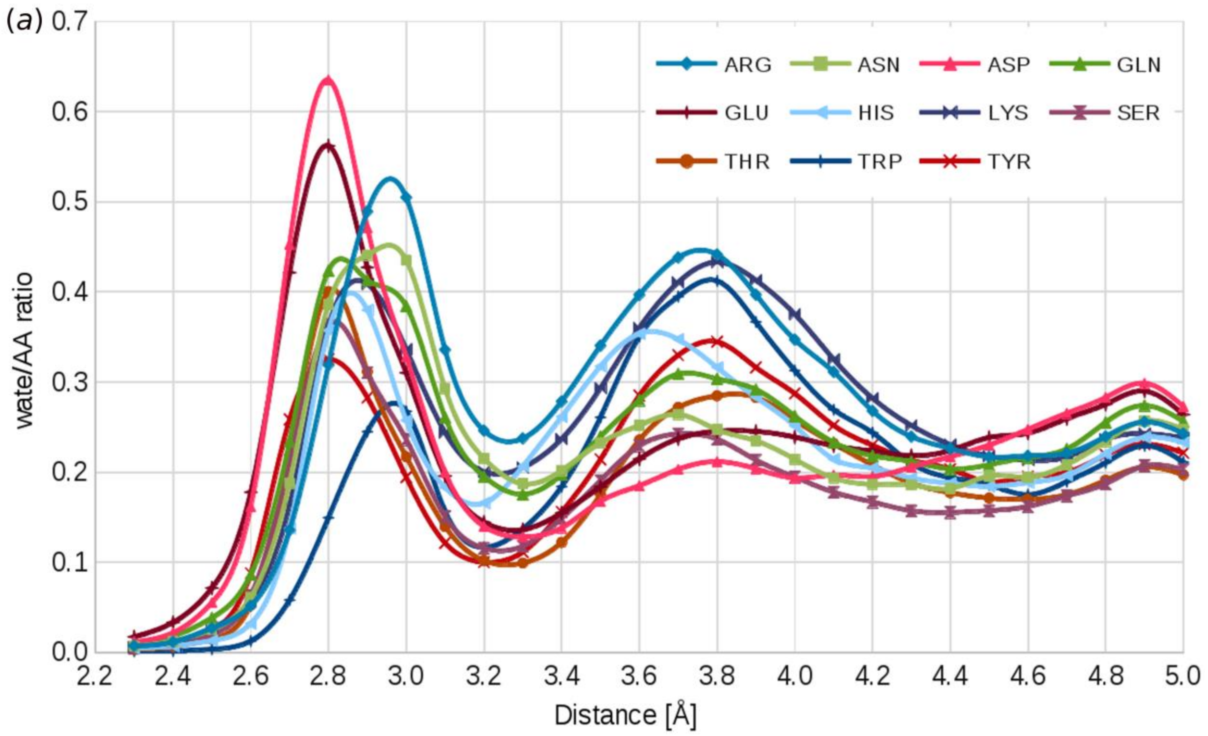

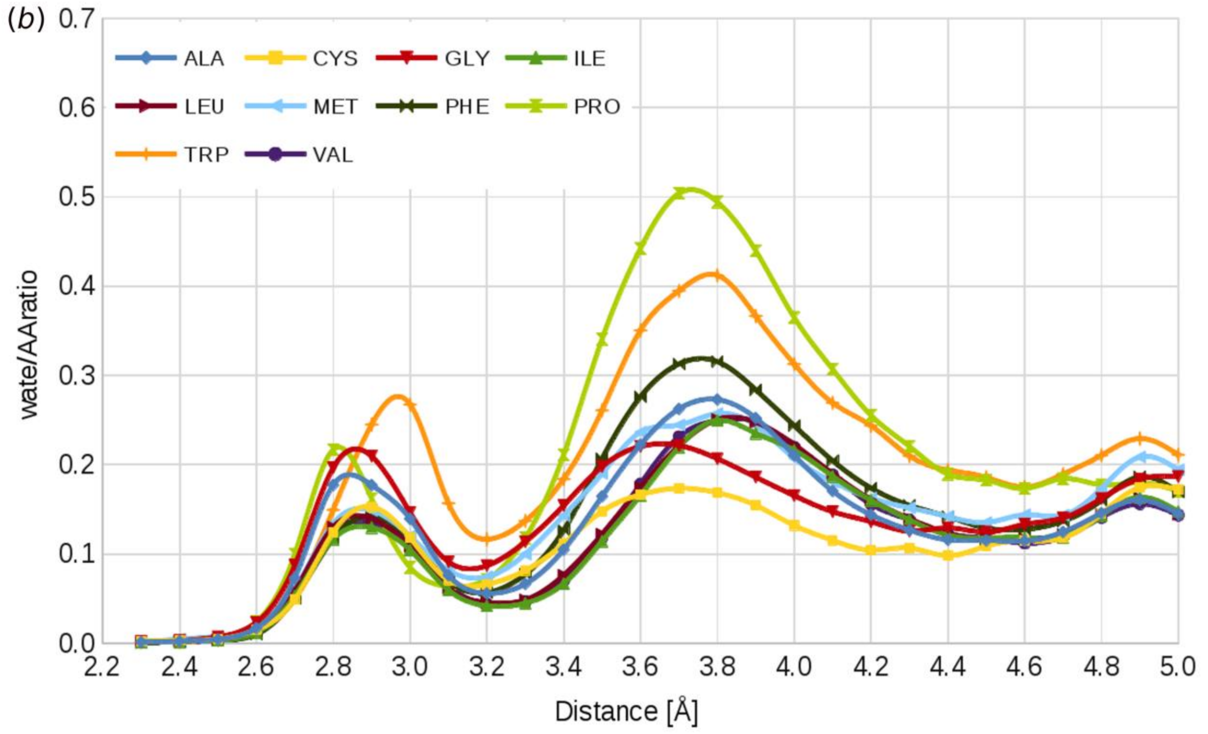

Figure S1 Distance distribution of waters around (*a*) polar residues and (*b*) hydrophobic residues. All waters with given distance (within 0.1Å shells) from any heavy atom of the residue of the given type in the set of analyzed protein chains are counted.

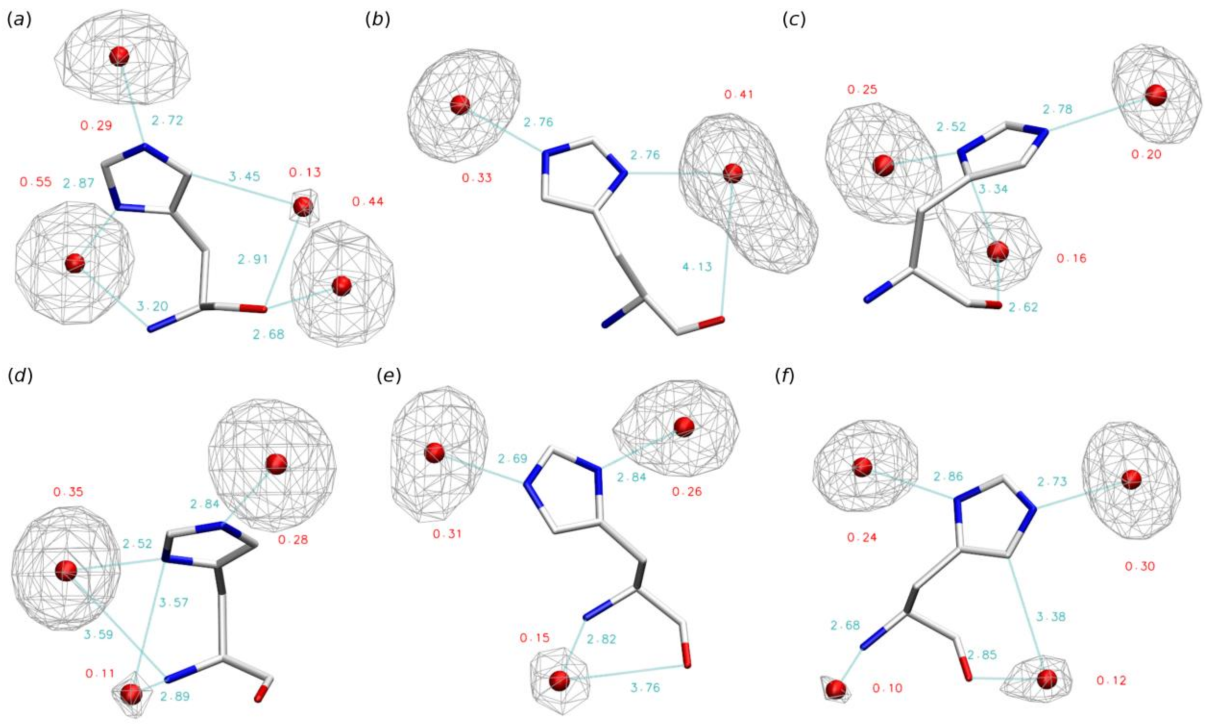

Figure S2 Hydration sites of His conformers. (*a*) His_H_g+, (*b*) His_H_g-, (*c*) His_H_t, (*d*) His_E_g+, (*e*) His_E_g-, (*f*) His_E_t. Positions of HS are shown as spheres and their occupancies and distances to nearest polar atom are labeled. Water distributions are contoured at occupancy level 0.10 using a mesh.

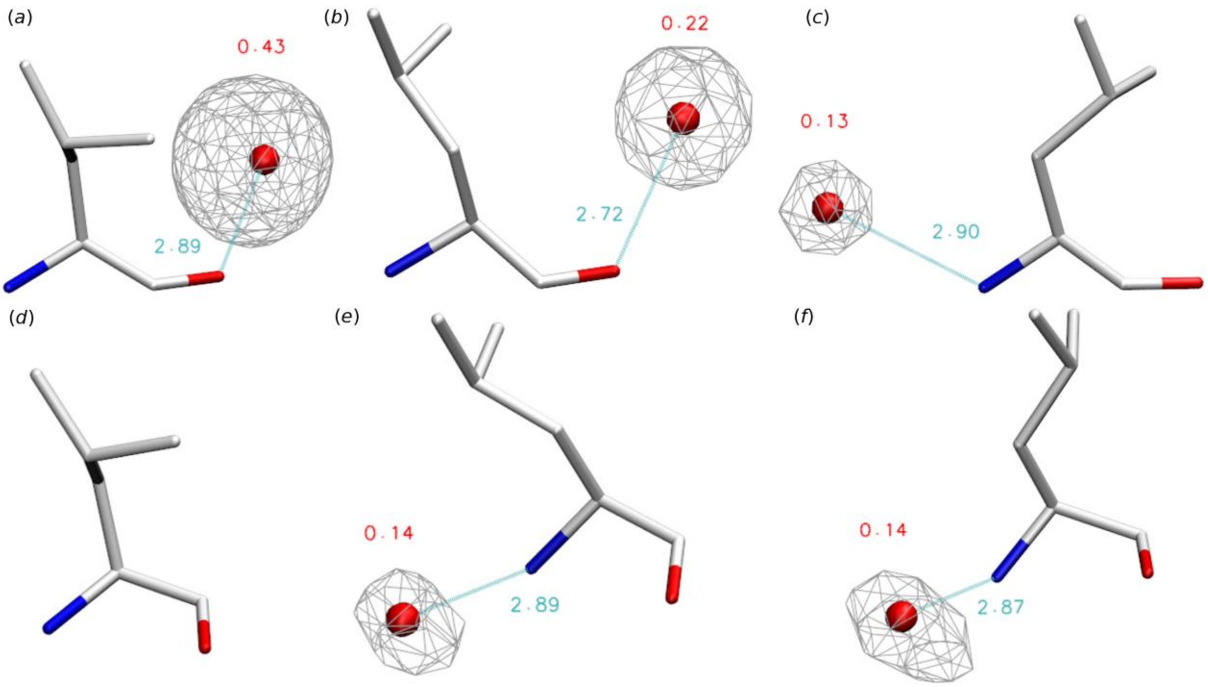

Figure S3 Hydration sites of Leu conformers. (*a*) Leu_H_g+, (*b*) Leu_H_g-, (*c*) Leu_H_t, (*d*) Leu_E_g+, (*e*) Leu_E_g-, (*f*) Leu_E_t. Positions of HS are shown as spheres and their occupancies and distances to nearest polar atom are labeled. Water distributions are contoured at occupancy level 0.10 using a mesh.

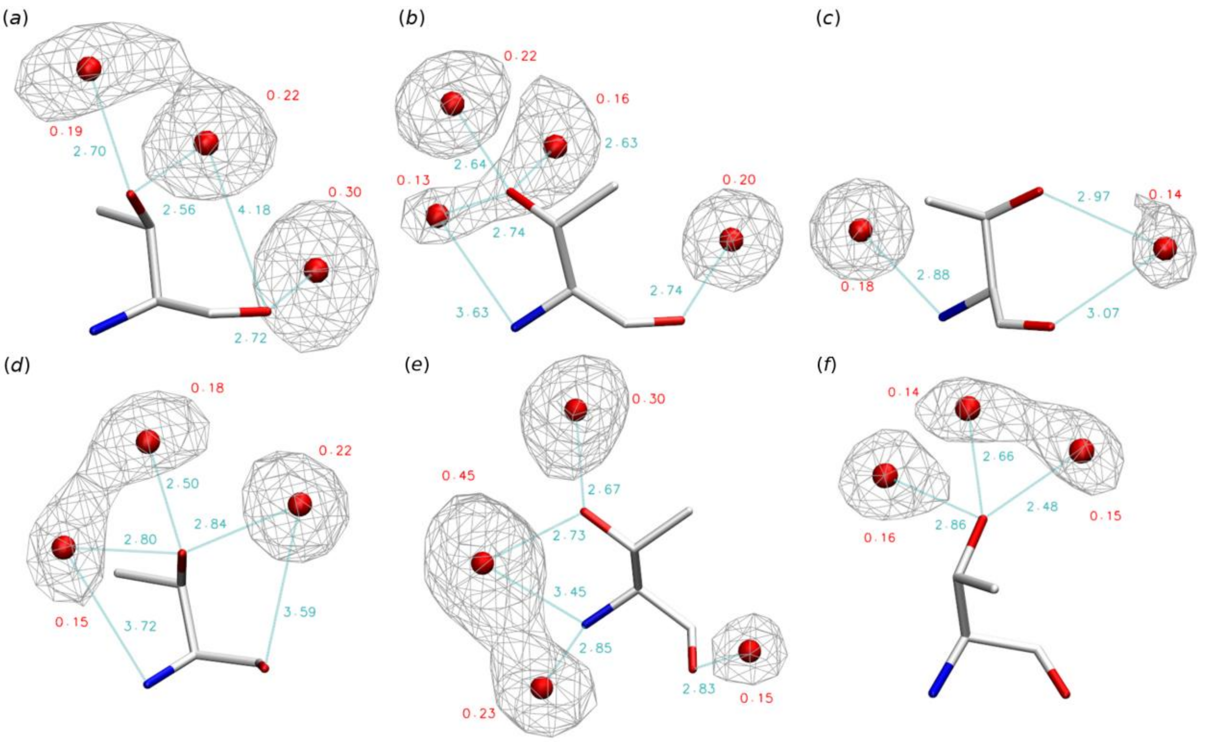

Figure S4 Hydration sites of Thr conformers. (*a*) Thr_H_g+, (*b*) Thr_H_g-, (*c*) Thr_H_t, (*d*) Thr_E_g+, (*e*) Thr_E_g-, (*f*) Thr_E_t. Positions of HS are shown as spheres and their occupancies and distances to nearest polar atom are labeled. Water distributions are contoured at occupancy level 0.10 using a mesh.

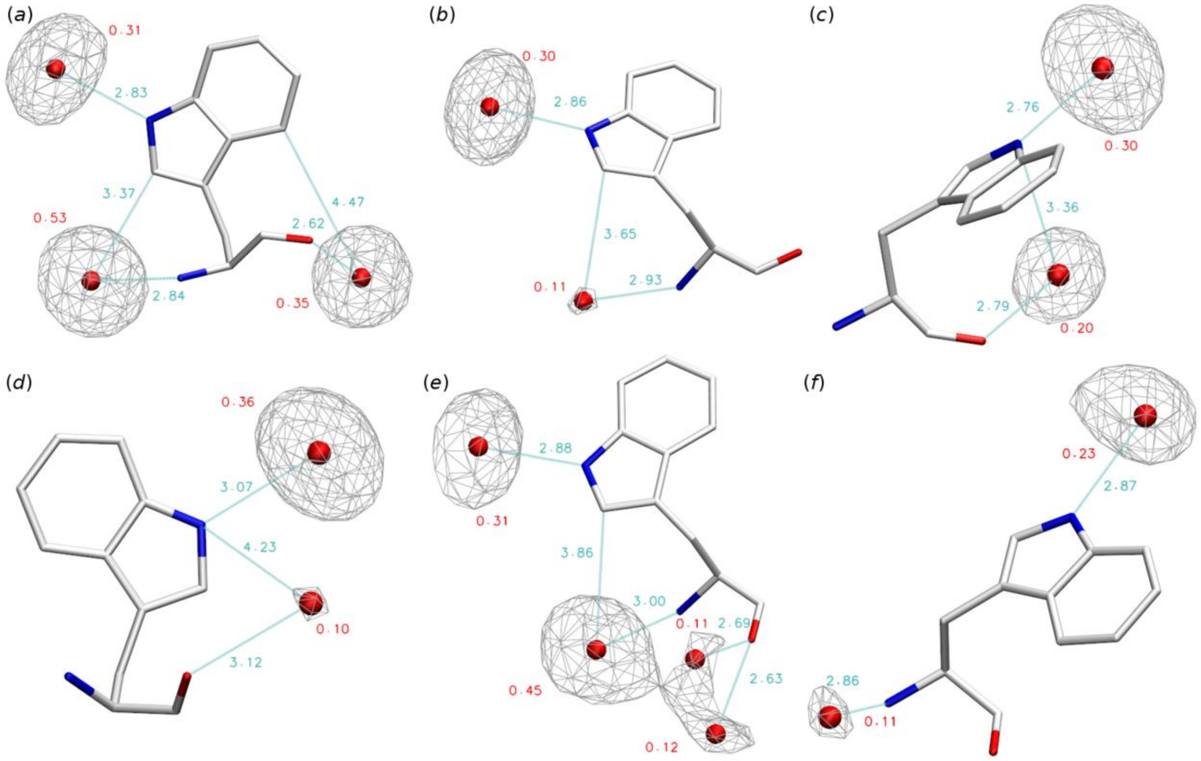

Figure S5 Hydration sites of Trp conformers. (*a*) Trp_H_g+, (*b*) Trp_H_g-, (*c*) Trp_H_t, (*d*) Trp_E_g+, (*e*) Trp_E_g-, (*f*) Trp_E_t. Positions of HS are shown as spheres and their occupancies and distances to nearest polar atom are labeled. Water distributions are contoured at occupancy level 0.10 using a mesh.

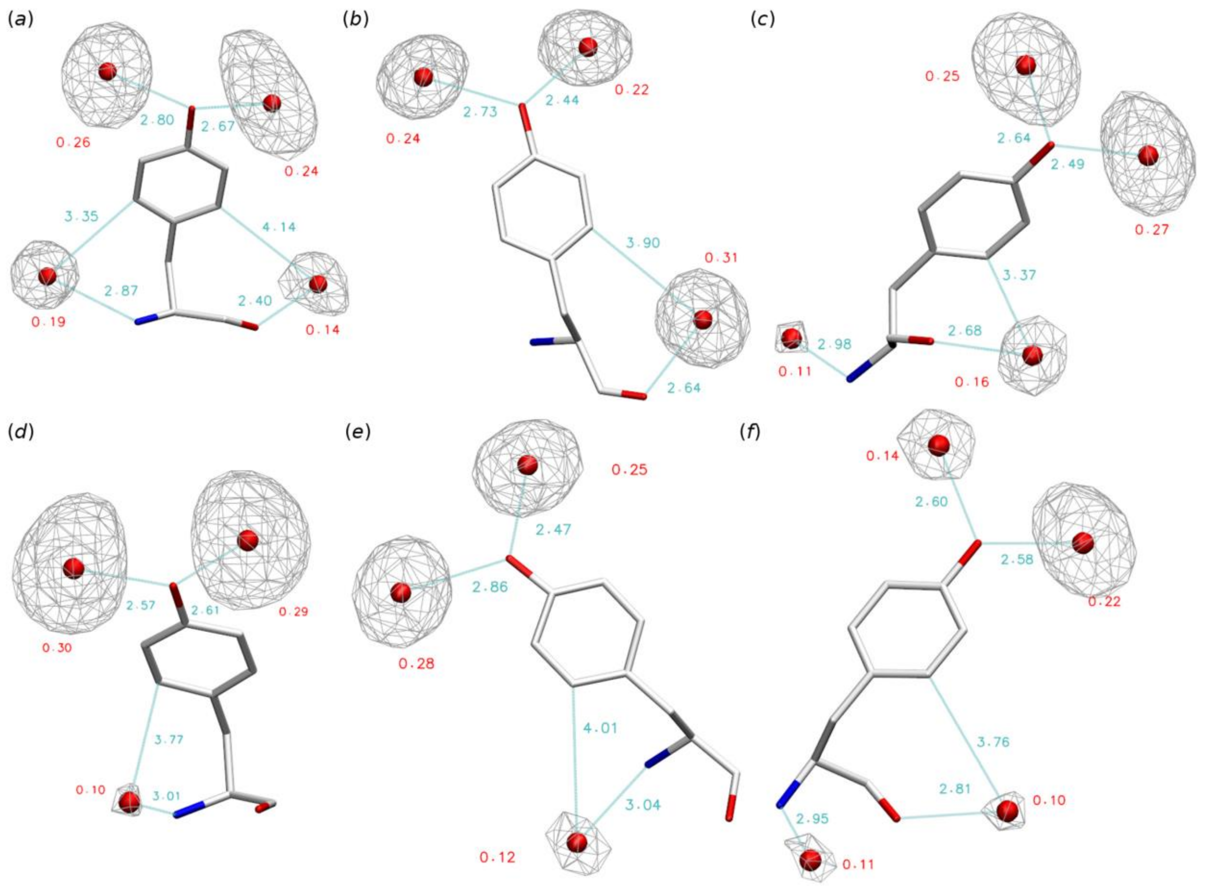

Figure S6 Hydration sites of Tyr conformers. (*a*) Tyr_H_g+, (*b*) Tyr_H_g-, (*c*) Tyr_H_t, (*d*) Tyr_E_g+, (*e*) Tyr_E_g-, (*f*) Tyr_E_t. Positions of HS are shown as spheres and their occupancies and distances to nearest polar atom are labeled. Water distributions are contoured at occupancy level 0.10 using a mesh.
